# Improvement in Primary Autoimmune Myelofibrosis Following a Short Course of Steroids and Intravenous Immunoglobulins

**DOI:** 10.7759/cureus.29735

**Published:** 2022-09-29

**Authors:** Swathi Prakash, Sara Alhariri, Mariam Hassan, Priya K Patel, Javier Corral

**Affiliations:** 1 Internal Medicine, Texas Tech University Health Sciences Center El Paso, El Paso, USA; 2 Hematology and Oncology, Texas Tech University Health Sciences Center El Paso, El Paso, USA

**Keywords:** intravenous immunoglobulin (ivig), fibrosis, primary aimf, aimf, ivig, steroids, myelofibrosis, bone marrow fibrosis, autoimmune disease, autoimmune myelofibrosis

## Abstract

Bone marrow fibrosis (BMF) is a histopathological finding appreciated in a multitude of conditions such as myeloproliferative diseases and malignant neoplasms, along with autoimmune disorders. Autoimmune myelofibrosis (AIMF) is a particularly uncommon etiology of benign BMF. AIMF may be primary with serologic evidence of autoantibodies or secondary to an underlying autoimmune disease. The authors aim to emphasize the importance of distinguishing between primary versus secondary causes owing to significant prognostic and therapeutic discrepancies and in hopes of expediting the diagnostic journey. Research has recommended a treatment strategy of high-dose steroids followed by a steroid taper. However, our patient responded positively to a short course of high-dose steroids and intravenous immunoglobulins (IVIG) as evidenced by an improvement in cytopenias and bone marrow fibrosis grading. This outcome warrants further research on the necessity of steroid tapers in AIMF.

## Introduction

Autoimmune myelofibrosis (AIMF) is a rare, underrecognized pathological entity that can present as a primary disease known as primary AIMF or secondary to an established systemic autoimmune disorder, with most cases affecting females before the age of 40 [1,2]. These autoimmune conditions include, but are not limited to, systemic lupus erythematosus (SLE), rheumatoid arthritis, Sjögren’s syndrome, ulcerative colitis, and primary biliary cirrhosis [3-5]. A single-institution, retrospective chart review further solidified that a majority of patients diagnosed with AIMF have concomitant autoimmune disorders [1,3].

AIMF will exhibit bone marrow reticulin fibrosis on bone marrow biopsy (BMP), leading to leukoerythroblastic anemia, thrombocytopenia, mild teardrop poikilocytosis, autoantibody production that is unrelated to autoimmune disease, and hypocomplementemia [1,2]. An update on 29 cases highlighted that primary AIMF and secondary autoimmune myelofibrosis may be difficult to distinguish, except for an increased incidence of granulocytic hyperplasia in primary AIMF [6]. The absence of splenomegaly, eosinophilia, basophilia, and the absence of abnormal myeloid, erythroid, or megakaryocytic morphology can help rule out myelofibrosis secondary to myeloproliferative disease [3].

While AIMF responds exceptionally to a short course of corticosteroids, secondary autoimmune myelofibrosis is typically treated with a combination of therapies targeting the myelofibrosis as well as the inciting myeloproliferative disorder, such as polycythemia vera or essential thrombocythemia. This further accentuates the importance of recognizing a culprit autoimmune disorder, if present, and carefully investigating the clinical features, laboratory markers, and idiosyncratic morphology of AIMF. The authors intend to provoke a high clinical suspicion of AIMF.

## Case presentation

A 47-year-old female presented with generalized weakness and easy bruising of five months’ duration. The patient was notably tachycardic (136 beats per minute) with multiple ecchymotic skin lesions involving the back and upper extremities and without hepatosplenomegaly. Laboratory workup was significant for hemoglobin of 6.8 g/dL, platelets of 21,000/µL, and an erythrocyte sedimentation rate of 139 mm/hour. Hematological studies revealed immunoglobulin G (IgG)-mediated autoimmune hemolytic anemia (positive direct antiglobulin test (DAT)) with a low reticulocyte index.

**Figure 1:**
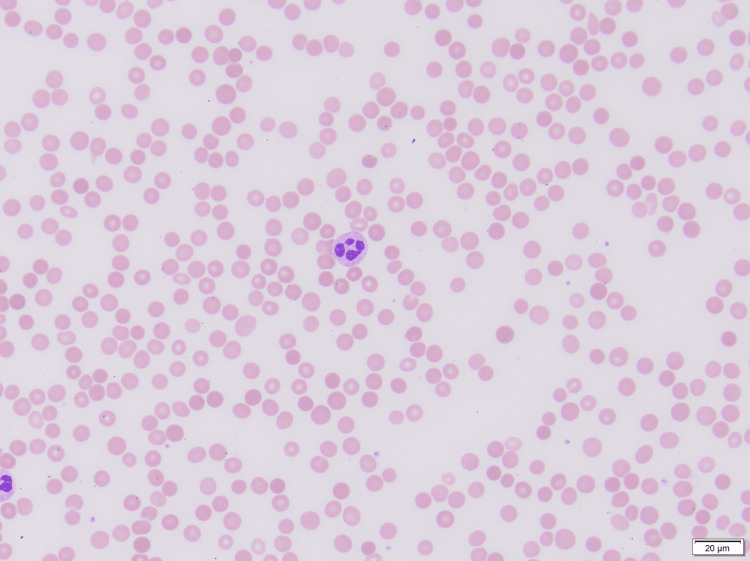
Peripheral blood smear showing spherocytes and thrombocytopenia (Diff-Quik stain, 400×)

**Figure 2:**
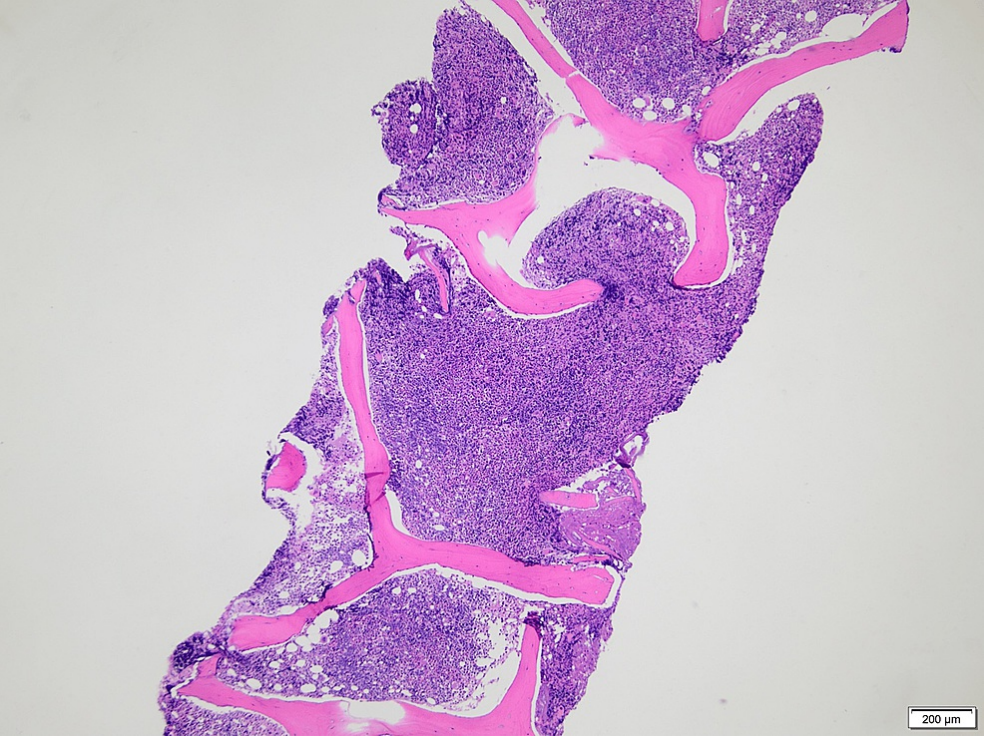
Bone marrow biopsy showing hypercellular marrow with greater than 95% cellularity (hematoxylin and eosin-stained section, 40×)

**Figure 3:**
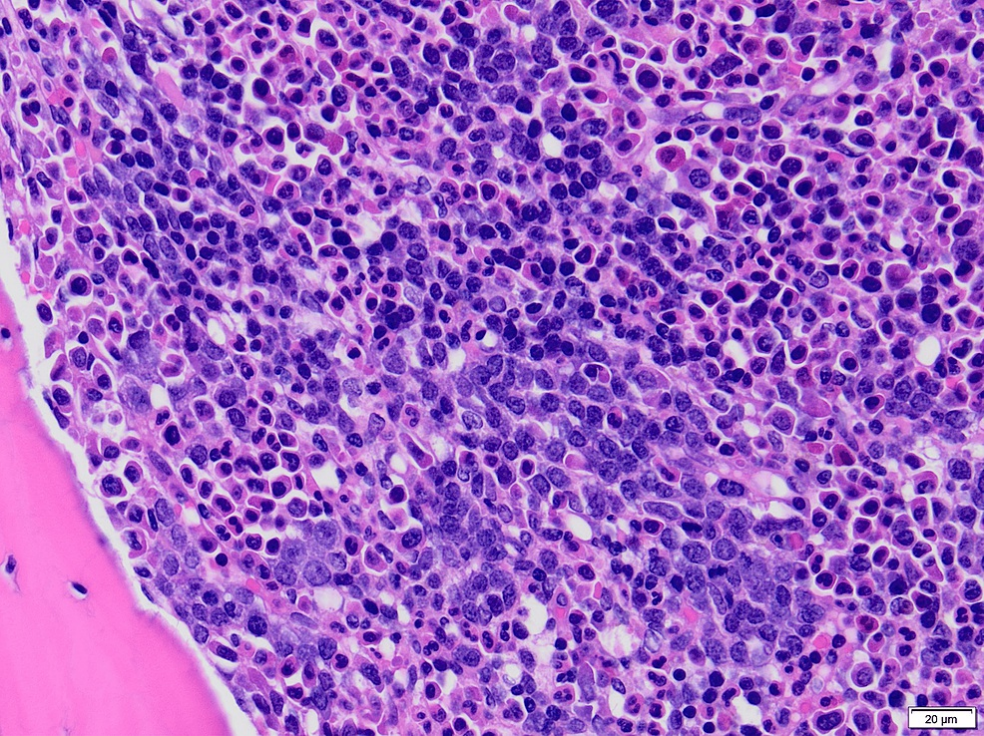
Bone marrow biopsy showing increased erythroid precursors including clusters of erythroblasts confirmed by positive E-cadherin stain (hematoxylin and eosin-stained section, 400×; E-cadherin not shown)

Prior hospitalization findings included pancytopenia for which she underwent extensive evaluation, including a bone marrow biopsy revealing erythroid hyperplasia and myelofibrosis (MF2-3), which was unlikely to be secondary to myelodysplastic syndrome. An autoimmune workup including antinuclear antibody (ANA), serum protein electrophoresis (SPEP), urine protein electrophoresis (UPEP), anti-double-stranded DNA (dsDNA), myeloperoxidase antibodies (MPO Ab), cyclic citrullinated peptide antibodies (CCP Ab), and rheumatoid factor (RF) was all negative, except for atypical perinuclear antineutrophil cytoplasmic antibodies (p-ANCA) titers of 1:80. The patient was also tested for infectious etiologies and was found to have a positive Epstein-Barr virus immunoglobulin G (EBV IgG), although its significance was unknown. Peripheral blood smear showed normocytic anemia with spherocytes and thrombocytopenia in the absence of blasts as depicted in Figure 1. Serum mutation analysis was negative for Janus kinase 2 (JAK2), myeloproliferative leukemia (MPL) protein, and calreticulin (*CALR*) gene mutations. Bone marrow biopsy displayed a markedly hypercellular (>90%), granulocytic hyperplasia with full maturation, left-shifted erythroid hyperplasia, and reticulin fibrosis (moderate to marked), along with the presence of megakaryocytes as depicted in Figure 2 and Figure 3. Bone marrow cytogenetic analysis was negative for genetic mutations.

**Figure 4:**
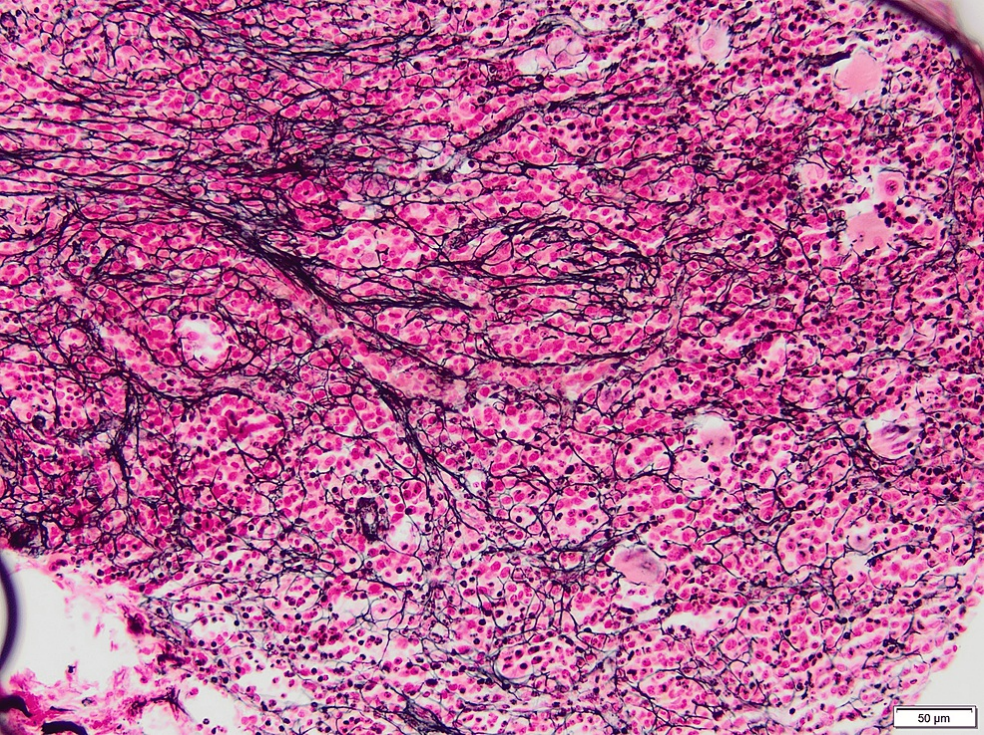
Reticulin stain showing myelofibrosis, MF1-2 (200×, bottom right)

The patient was treated with intravenous immunoglobulin (IVIG) for two days and five days of prednisone at 1 mg/kg. Post-treatment bone marrow biopsy was suggestive of idiopathic myelofibrosis (MF1-2 reticulin stain) as depicted in Figure 4. Immunohistochemistry was positive for CD3, CD20, CD113, CD34, E-cadherin, CD79a, and MPO. Clinical and hematological parameters subsequently improved. The patient’s hemoglobin increased to 9.7 g/dL, and the patient had a platelet count of 228,000/µL upon discharge. The plan was to continue on a steroid taper on an outpatient basis, but the patient was lost to follow-up.

## Discussion

Bone marrow fibrosis (BMF) is defined as the increased deposition of reticulin fibers, with or without collagen fibers, in the bone marrow [7]. Elevated pro-inflammatory cytokines, particularly transforming growth factor-beta (TGF-β), provide a potent stimulant for fibroblasts to generate the extracellular matrix (ECM) while simultaneously upregulating lysyl oxidase (which stabilizes the ECM) and inhibiting the enzymes that degrade the ECM [7]. The dysregulation of Janus kinase 2-signal transducers and activators of transcription (JAK2-STAT) is highly implicated in the pathogenesis of BMF [7]. BMF can be secondary to malignancies (myeloproliferative neoplasms (MPNs), metastatic solid tumors, and acute megakaryocytic leukemia), chronic infections (HIV and tuberculosis), adverse drug reactions, autoimmune conditions (SLE and Sjogren’s syndrome), or AIMF [7].

AIMF is a rare disease characterized by isolated or combined cytopenias with BMF noted in conjunction with autoimmune diseases [1]. There are two types of AIMF: a primary disease characterized by patients having an autoimmune disease without serologic evidence of autoantibodies and secondary AIMF in the setting of an underlying autoimmune process [8]. Secondary AIMF is more common, and SLE is the most common instigator, contributing to up to 72% of cases [8]. In primary AIMF, the most common presentation is a positive direct antiglobulin test (DAT) with or without autoimmune antibodies [8].

A review published in the American Journal of Clinical Pathology divulges how three patients were diagnosed with AIMF by screening for SLE using criteria from the American Rheumatism Association [2,8]. There was no serologic evidence for SLE, although the patients exhibited symptoms of synovitis with polyclonal hypergammaglobulinemia, psoriatic arthritis, a positive direct antiglobulin test (DAT), and hemolytic anemia [2]. Additional physical examination findings associated with AIMF may include malar rash, oral ulcers, pleuritis, pericarditis, hepatosplenomegaly, and lymphadenopathy; these, however, significantly overlap with SLE and other autoimmune conditions [2].

AIMF is commonly mistaken for primary myelofibrosis (PMF). The key differentiating factor is that PMF is a myeloproliferative neoplasm characterized by stem cell-derived clonal myeloproliferation associated with JAK2, CALR, or MPL mutations [9]. In AIMF, clonality and mutations such as JAK2, CALR, and MPL mutations are absent [6]. The absence of marked teardrop poikilocytosis and immature myeloid precursor cells in circulation may also aid in differentiating AIMF from primary myelofibrosis [8]. It is imperative to be able to recognize and diagnose the different conditions as their treatments are vastly different.

In a paper published by Piatek et al., the features that favored AIMF over PMF were the decrease or complete absence of leukoerythroblastic reactions in the peripheral smear, absence of basophilia or eosinophilia, mild degree of BMF, absence of osteosclerosis, hypercellular marrow, presence of lymphoid aggregates, and absence of dysplasia [3]. A paper published by Pullarkat et al. in 2003 delineated the features of primary AIMF, which included a positive direct antiglobulin test (DAT) with or without autoimmune hemolytic anemia, presence of autoimmune phenomena, absence of splenomegaly, mild teardrop poikilocytosis, absence of clustered and dysmorphic megakaryocytes and osteosclerosis in bone marrow biopsy, presence of interstitial lymphocyte infiltration or lymphoid aggregates in bone marrow biopsy, and a positive response to corticosteroid therapy [8]. Although there are inconclusive and inconsistent diagnostic criteria for AIMF, there are several features reported across various pieces of literature, which could possibly help in diagnosing AIMF as described above.

The authors report the unusual case of a patient who demonstrated atypical p-ANCA titers in the ratio of 1:80. It is not suggestive of any autoimmune disease, although it does depict that antibodies are present. It is known that high p-ANCA titers of ≥1:80 are indicative of systemic autoimmune rheumatology diseases (SARDs) [10].

AIMF patients typically have a good clinical response to a course of steroids such as prednisone starting at 1 mg/kg and tapered over 1-3 months, resulting in complete normalization of peripheral blood counts in six out of seven patients [8]. Similarly, a case reported in 2020 involving a 66-year-old female with SLE-associated AIMF showed improvement in cytopenia after one month of oral corticosteroid therapy on bone marrow examination [11]. Our patient responded well to a short course of high-dose steroids and IVIG, with a significant improvement in cytopenias and bone marrow fibrosis grading from MF 2-3 to MF 1-2. Although a steroid taper may not be necessary in every patient such as our patient, it may however be required in some cases to prevent relapse of primary AIMF or prevent an exacerbation of an underlying autoimmune disease if present. Further research needs to be conducted on the necessity of a steroid taper in primary AIMF.

For steroid-refractory patients, intravenous gamma globulin, cyclophosphamide, cyclosporine, danazol, colchicine, vincristine, or azathioprine can lead to improvement of cytopenias and fibrosis regression [7,8]. Our patient responded to a combination of steroids and IVIG with a significant improvement in cytopenias and improvement in myelofibrosis grading of MF2-3 to MF1-2 on bone marrow biopsy examination. It is noted that in AIMF, corticosteroids, and immunosuppressants often lead to regression of BMF [10].

A Swedish study found that patients with autoimmune diseases such as Crohn’s disease, polymyalgia rheumatica, and aplastic anemia had a 20% higher risk of developing myeloproliferative neoplasms (MPNs) than those without any autoimmune disease [10]. This is attributed to a multitude of factors, including environmental and genetic. Inflammation, specifically, may cause neoplastic disease by altering the cellular composition of the bone marrow [10]. A study of 32 patients with AIMF reported that 29 patients showed marked improvement in cytopenias with corticosteroid treatment, whereas the three patients who were minimally affected or unaffected by corticosteroids improved with immunosuppressants such as azathioprine, cyclophosphamide, or IVIG [12]. Thus, immunosuppressive therapies may improve fibrotic damage in the bone marrow when corticosteroid therapy is inadequate.

On the other hand, some patients can be refractory to steroids, and one case report revealed that a 61-year-old female with primary AIMF did not respond to a prednisone taper of 1 mg/kg for three months, nor did the patient respond to rituximab, danazol, or cyclosporine. She did, however, show marked improvement with the Janus kinase (JAK1/JAK2) inhibitor ruxolitinib with the improvement of marrow fibrosis from severe (MF 3/3) to mild (MF 1/3), suggesting that other immunosuppressants can play a role in the improvement of cytopenia, transfusion dependency, and marrow fibrosis [13].

The current standard of treatment for myelofibrosis consists of JAK inhibitors. Patients may experience spleen size reduction and symptom relief, but inhibiting the JAK receptor does not delay the progression of the disease [14]. The Controlled Myelofibrosis Study With Oral Janus-associated Kinase Inhibitor Treatment-II (COMFORT-II) trial studied the effects of ruxolitinib versus the best available therapy in patients with myelofibrosis. The analysis showed that there were durable benefits to patients in terms of spleen volume reductions, symptom relief, and reductions in JAK2 V617F allele burden, and a good proportion of patients had maintained or improved their BMF, which was notable in long-term ruxolitinib use [15]. The side effects of this treatment include potential worsening of cytopenias, rendering patients with coexisting cytopenias ineligible for this treatment [15]. Hence, therapies that do not involve inhibiting the JAK receptor are currently being explored, such as treatment with anti-fibrosing agents and immunomodulators.

One such anti-fibrosing agent is simtuzumab, a humanized antibody against the lysyl oxidase-like (LOXL) enzyme. LOXL is a catalytic agent that allows for cross-linking between collagen and elastin, the underlying mechanism for fibrotic disease. Preclinical studies have shown that the inhibition of LOXL may lead to improvement in BMF [16]. Although simtuzumab was well-tolerated by patients in a phase II study, there was no significant reduction in BMF [16].

PRM-151, another anti-fibrosing agent, has shown more success than simtuzumab in treating myelofibrosis. PRM is a pentraxin 2 recombinant that was initially used in pulmonary fibrosis with the aim to regulate the differentiation of monocytes into fibrocytes and pro-fibrotic macrophages at sites of tissue damage [16]. A phase II study of PRM used in combination with ruxolitinib resulted in 35% of patients experiencing regression of BMF, 40% experiencing improvement in cytopenia, and 26% experiencing a reduction in spleen size [16]. Although there have been significant advances in the treatment of bone marrow fibrosis, further research needs to be conducted on patients who have autoimmune myelofibrosis.

## Conclusions

This is a unique case report describing the improvement of myelofibrosis after steroid treatment for primary AIMF. Since AIMF is a benign condition with a positive prognosis, it is imperative to identify this uncommon cause of BMF to allow for a more favorable clinical course with corticosteroid therapy. Although patients with AIMF usually present with non-clonal bone marrow morphology and autoantibodies, patients with primary AIMF may also present with these findings, complicating the distinction between the two in a clinical setting. The marked clinical, hematological, and histological improvement in disease following a short course of steroids paves way for further research and possibly treatment guidelines in the management of this rare disorder.
